# Heavy metal exposure reverses genetic resistance to *Chlamydia*-induced arthritis

**DOI:** 10.1186/ar2610

**Published:** 2009-02-09

**Authors:** Robert D Inman, Basil Chiu

**Affiliations:** 1 Division of Genes and Development, Toronto Western Research Institute, 399 Bathurst Street, Toronto, ON, M5T 2S8, Canada; 2 Department of Medicine, University of Toronto, 190 Elizabeth Street, Toronto, ON, M5G 2C4, Canada

## Abstract

**Introduction:**

We have previously observed that Brown Norway (BN) rats display a relative resistance to experimental *Chlamydia*-induced arthritis. In the present study, we examine an environmental toxin, mercuric chloride (HgCl_2_), as a modulator of this innate resistance to arthritis.

**Methods:**

To assess the effect of the heavy metal exposure, one group of rats received two subcutaneous injections of HgCl_2 _(1 mg/kg) 48 hours apart. Seven days later, the animals received the intra-articular injection of synoviocyte-packaged *Chlamydia*.

**Results:**

Histopathology revealed that BN rats receiving only *Chlamydia *had a minimal cellular infiltration in the joint, which was predominantly mononuclear in character. In contrast, mercury-exposed rats had a marked exacerbation of the histopathological severity of the arthritis, and the infiltration was predominantly neutrophilic. Mercury exposure was also associated with marked enhancement in IgE levels and an alteration in IgG2a/IgG1 ratio, reflecting a Th2 shift. The local cytokine profile in the joint was markedly altered after mercury exposure, with a suppression of tumour necrosis factor-alpha and interferon-gamma but an enhancement of vascular endothelial growth factor. This was associated with decreased host clearance capacity reflected in enhanced bacterial load in both the spleen and the joint and was accompanied by enhanced detection of microbial antigens in the synovial tissues by immunohistological staining.

**Conclusions:**

Genetically defined cytokine production in the joint defines the severity of reactive arthritis by dictating the local clearance of the pathogen. This interplay can be altered dramatically by heavy metal exposure, which results in suppression of protective cytokines in the microenvironment of the joint.

## Introduction

Many rheumatic diseases are thought to reflect an interplay of genetic susceptibility and environmental triggers, but there are few examples in which strong clues for the nature of these interacting elements are known. One such example is reactive arthritis (ReA), in which susceptible individuals develop an aseptic arthritis following an extra-articular infection. Yet dissecting the immune mechanisms in ReA has proven to be difficult in the clinical setting. Experimental *Chlamydia trachomatis*-induced arthritis (CtIA) affords the opportunity to systematically address the factors that define outcomes after exposure to this arthritogenic pathogen [1]. We have recently observed that the Brown Norway (BN) rat is resistant to CtIA [2], with minimal transient inflammation in the joint. The infiltrating cell population in the joint is primarily mononuclear in nature and joint damage is minimal. This contrasts with an aggressive neutrophilic infiltration with associated joint injury that is seen in susceptible strains.

In contrast to their inherent resistance to this arthritis, BN rats are uniquely susceptible to the development of a variety of autoimmune conditions mediated mainly through Th2 mechanisms [3]. One such condition is the development of autoimmunity subsequent to an exposure of mercury [4]. The hallmarks of this condition are the increase of circulating IgE, the overproduction of interleukin-4 (IL-4), and the activation of Th2 cells [5,6]. The normal CD4/CD8 balance and their associated cytokines are markedly disrupted [7]. This is accompanied by the appearance of a range of autoantibodies [5,8,9]. Early pivotal studies examining the effects of mercuric chloride (HgCl_2_) in rats documented a significant increase in total IgE and this was observed in BN rats but not in Lewis rats [10]. Subsequent studies revealed that HgCl_2 _induction of IL-4 was accompanied by a decrease in CD23 expression on B cells and that IL-2, IL-6, and IL-10 were upregulated as well as IL-4 following HgCl_2 _exposure in BN rats [11,12]. Because the BN rat displays a dichotomy of resistance to infection-triggered arthritis but susceptibility to mercury-induced immune disruption, we addressed how these factors would interact if they were temporally related in the rat.

## Materials and methods

### Animals

Eight week-old male BN rats were purchased from Harlan Laboratories, Inc. (Indianapolis, IN, USA). The animals were maintained in microisolators under specific pathogen-free conditions in the animal care facility of the Toronto Western Hospital. All animals studied were less than 12 weeks of age. The studies were conducted with the approval of the Animal Care Committee of the University Health Network.

### Induction of arthritis

#### *Chlamydia trachomatis*-induced arthritis

Arthritis was induced in the rats by intra-articular injection of synoviocyte-packaged *Chlamydia *as described [2]. Briefly, *C. trachomatis *serotype L2 was inoculated onto monolayers of rat synovial fibroblasts (SFs) in tissue culture. These stable SF lines were developed as described [1]. After overnight incubation, cells were harvested and adjusted to 5 × 10 PP^5PP^/mL. Rats were anaesthetized with isoflurane (Pharmaceutical Partners of Canada, Richmond Hill, ON, Canada), and 0.2 mL of the infected cells containing 2 × 10 PP^5PP ^colony-forming units (CFU) of *Chlamydia *was injected into the knee joint. Joint swelling was measured with a caliper and recorded in millimetres. Animals were euthanized at day 7 post-injection. Mock injections on non-infected synoviocytes had previously been shown to induce only a transient inflammation in the joint.

### Protocol and injection schedule for HgCl_2_

Several different dose schedules have been used previously by investigators to address response to HgCl_2 _[5,6,8,9]. Roos and colleagues [13,14] used a two-injection abbreviated schedule to investigate the short-term effects of HgCl_2 _exposure. This protocol was associated with less morbidity than other protocols using five injections over a 10-day period. These investigators observed that distinctive immunological changes were rapid and were observed as early as 4 days after the initial HgCl_2 _exposure, with minimal toxicity. We therefore adopted the two-injection protocol of Roos and colleagues [13,14]. HgCl_2 _(Sigma-Aldrich, St. Louis, MO, USA) was dissolved in water at 1 mg/mL and then filter-sterilized. The rats were anaesthetized by inhalation using isoflurane, weighed, and injected with the HgCl_2 _solution subcutaneously at a dosage of 1 mg/kg of body weight. Two days later, the injection procedure was repeated. The animals tolerated this procedure well, with no local or systemic adverse effects noted. There was no joint swelling or joint discomfort noted following the two injections of HgCl_2_. Seven days after the second HgCl_2 _injection, the rats were anaesthetized again and synoviocyte-packaged *Chlamydia *(2 × 10^5 ^CFU) was delivered by intra-articular injection into the knee joint in accordance with our established protocol [2]. Control rats without antecedent exposure to HgCl_2 _received a similar intra-articular injection of synviocyte-packaged *Chlamydia*. One week after the injection of the joint, the animals were sacrificed by anaesthetic overdose. Eight animals were used for each comparative experimental condition.

### Processing and pathology

The knee joints were removed and fixed in formalin. The lateral width of each joint was measured with a caliper. After measurement, the joints were decalcified and processed for histopathological scoring as described [2]. Immunopathology studies used a primary antibody specific for *C. trachomatis *(AbD Serotec, Oxford, Oxfordshire, UK) that was developed with a peroxidase-conjugated anti-mouse antibody.

### *Chlamydia *quantitation

The Dako IDEIA™ *PCE Chlamydia *Kit (Dako, Ely, Cambridgeshire, UK) was used for determining the clearance of the pathogen. Seven days after the intra-articular injection of *Chlamydia*, the HgCl_2_-exposed and non-exposed rats were sacrificed. Spleens were removed and ground over a Falcon Cell Strainer (BD Biosciences 2280 Argentia Road, Mississauga, ON L5N 6H8, Canada) using the rubber end of a syringe in a Petri dish containing 10 mL of phosphate-buffered saline (PBS). The tissue homogenate suspensions were frozen at -70°C until tested. For analysis of synovial tissue, the injected joints were dissected and the tissues were removed. Tissues were stored in 1 mL of the transport buffer provided with the enzyme-linked immunosorbent assay (ELISA) kit and then were frozen as above.

### Serology

Blood was obtained from the rats at sacrifice by cardiac puncture. Sera were separated and frozen at -70°C until used. Results reflect serological changes 14 days after HgCl_2 _exposure and 7 days after onset of CtIA.

### Total serum IgE

Elevation in serum IgE is the hallmark of Hg-induced autoimmunity in the BN rat. The ELISA for total serum IgE was performed using an antibody pair kit from AbD Serotec in accordance with the protocol of the manufacturer. A mouse anti-rat IgE monoclonal antibody was used to coat the ELISA plate. All sera were diluted 1:100 and incubated in the wells for 90 minutes. This was followed by the second antibody, peroxidase-conjugated anti-rat kappa/lambda, which was used at a 1:2,000 dilution for a second 90-minute incubation. O-phenylenediamine (Sigma-Aldrich) was used for development, and the plates were read at 490 nm.

### Anti-*Chlamydia *antibodies

The anti-*Chlamydia *antibody ELISA followed our published methods [1]. ELISA plates were coated with a 10-μg/mL preparation of *Chlamydia *in a pH 9.6 carbonate-bicarbonate buffer overnight at 4°C. A single batch of plates was prepared and kept frozen. Rat sera were diluted 1:200 in PBS containing 1% bovine serum albumin (BSA) as blocking agent, and 0.2 mL was added to each well in triplicate. After 90 minutes of incubation at 37°C, plates were washed and peroxidase-conjugated antibodies were added. Peroxidase-conjugated goat anti-rat IgG1 and IgG2a secondary antibodies were obtained from Bethyl Laboratories, Inc. (Montgomery, TX, USA) and used at a 1:10,000 dilution. After a further 90-minute incubation at 37°C, the plates were washed and colour-developed with o-phenylenediamine (0.4 mg/mL) as substrate. Plates were read at 490 nm with an ELISA plate reader.

### Anti-collagen_(II) _antibodies

The ELISA kit for the rat anti-type II collagen antibodies was from Chondrex (Redmond, WA, USA) and followed the protocol of the manufacturer. Rat serum was run at a 1:200 dilution in the assay.

### Cytokines

Serum and synovial tissue cytokine levels in the Hg-exposed and non-exposed BN rats were assayed as previously described [2]. Rat interferon-gamma (IFN-γ), tumour necrosis factor-alpha (TNF-α), IL-4, and IL-10 were assayed by ELISA kits purchased from Pierce Endogen (Thermo Fisher Scientific Inc., Rockford, IL, USA). The ELISA for rat vascular endothelial growth factor (VEGF) was purchased from Bender MedSystems (Vienna, Austria).

### Nitric oxide

The kit to determine total nitric oxide (NO) concentrations was from R&D Systems (Minneapolis, MN, USA) (KGE001). This is a colorimetric assay using the Griess reaction with the entire procedure performed on an ELISA plate. The serum samples are filtered through 10,000-molecular weight cutoff spin filter units (Millipore Microcon YM-10; Millipore Corporation, Billerica, MA, USA) prior to the assay. Serum samples (0.5 mL in size) were loaded on top of the filter units and then centrifuged in Eppendorf centrifuge at a speed of 14,000 rotations per minute for 30 minutes at room temperature.

### Statistical analysis

The Student *t *test was used to compare statistical differences between groups.

## Results

### Acute inflammatory response

**Figure 1:**
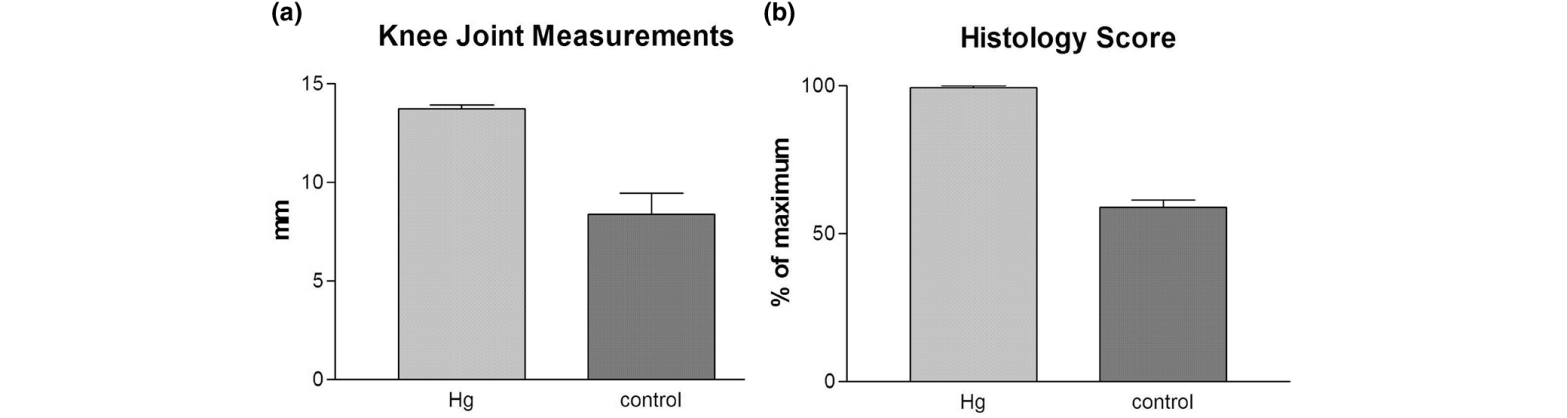
Joint swelling and histopathology scores demonstrating exacerbation of *Chlamydia*-induced arthritis following a prior systemic exposure to mercuric chloride (HgCl_2_) (n = 8 in each group). The differences after HgCl_2 _exposure are significant (*P *< 0.005) in both joint swelling **(a) **and histopathological severity scores **(b)**. Hg, mercury. Ct, *Chlamydia trachomatis*.

After induction of CtIA, a significant difference was observed between the HgCl_2_-exposed rats compared with controls. The rats with a prior exposure to HgCl_2 _demonstrated significantly more joint swelling compared with the normally mild swelling of naïve BN rats using the same injection protocol. The mean joint width in the HgCl_2_-exposed rats (n = 8) was 13.7 ± 0.53 mm in comparison with a control joint mean size (n = 8) of 9.6 ± 0.86 mm (*P *< 0.0001) (Figure 1a).

**Figure 2:**
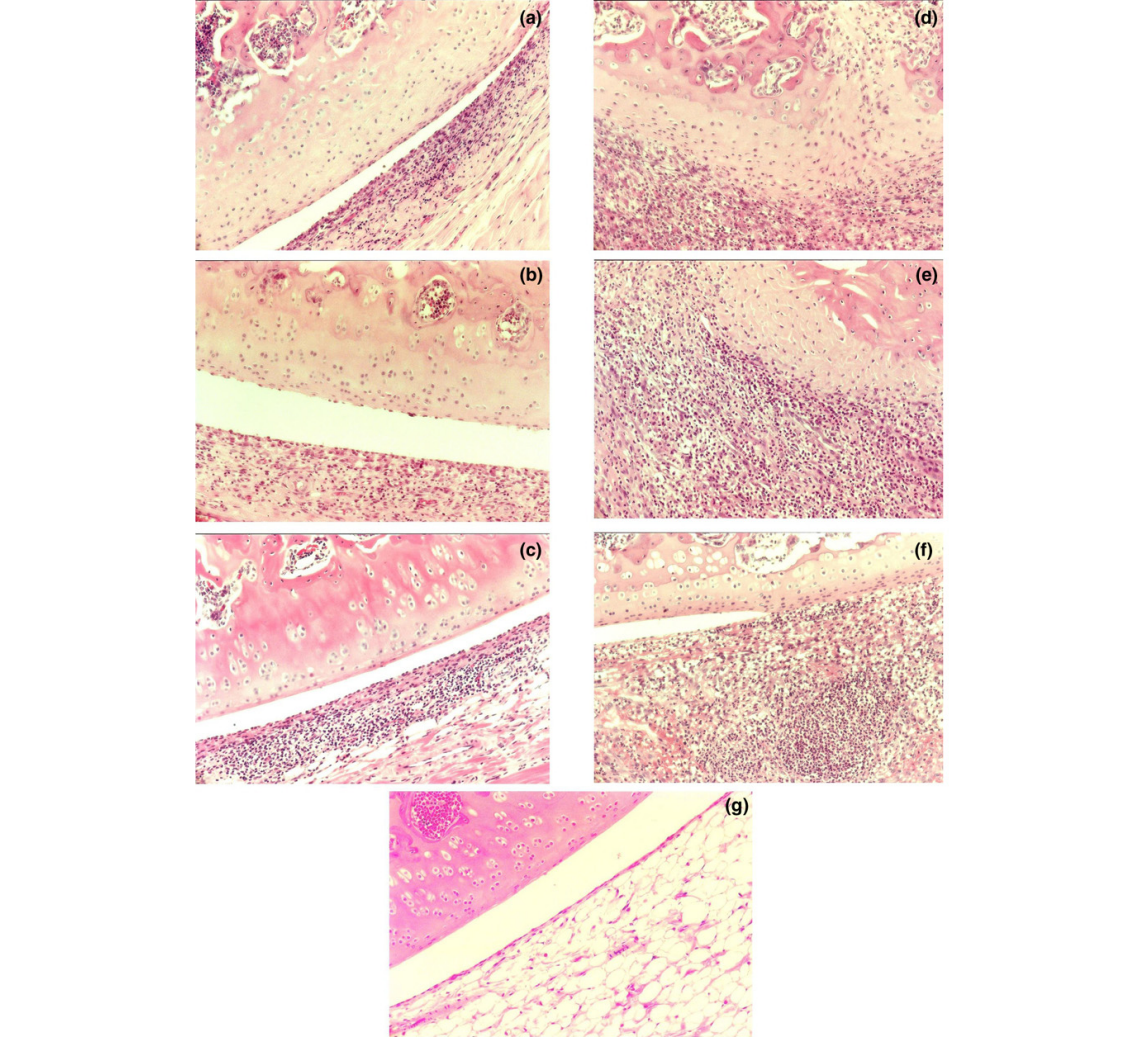
Histopathology (haematoxylin and eosin) in *Chlamydia trachomatis*-induced arthritis (CtIA) alone and after a prior exposure to mercuric chloride (HgCl_2_). CtIA in the non-HgCl_2_-exposed control rats is accompanied by only moderate synovial hypertrophy and hyperplasia and a mononuclear cell infiltration **(a-c)**. Eight animals were studied in each group. The joints of the HgCl_2_-exposed rats demonstrated more marked synovial hypertrophy and hyperplasia and a marked infiltration of the synovial tissues, primarily by neutrophils. This is accompanied by bone and cartilage destruction and by pannus formation, which invades subchondral bone **(d-f)**. Panel **(g) **is representative of a rat that received the HgCl_2 _injections alone and shows no pathological change in the joint. Original magnifications × 200.

Histologically, two systemic injections of HgCl_2 _induced no pathological changes in the joint (Figure 2). The CtIA in the non-HgCl_2_-exposed control rats was accompanied by only modest synovial hypertrophy and hyperplasia (Figure 2). Synovial tissue cellular infiltration was pleomorphic, with mononuclear cells comprising the dominant population. Pannus formation was minimal and there were only mild changes in bone and cartilage. This is the pattern observed in BN rats in our previous studies [2]. In contrast, the joints of the HgCl_2_-exposed rats demonstrated more marked synovial hypertrophy and hyperplasia (Figure 2). This was accompanied by massive infiltration of the synovial tissues with a predominance of neutrophils. There were areas of necrosis and bone and cartilage destruction by aggressive pannus formation. The invading pannus could be seen invading subchondral bone. Thus, the HgCl_2 _exposure altered the mild arthritis characteristic of resistant BN into the aggressive profile of the arthritis characteristic of susceptible rats. When the histopathological scoring system was applied, the joint scores of the HgCl_2_-exposed test rats (n = 8) were significantly higher (average score of 99.28% ± 1.89% of maximum) than those of controls (average score of 58.75% ± 7.44% of maximum) (*P *< 0.0001) (Figure 1b).

### Serum IgE

**Figure 3:**
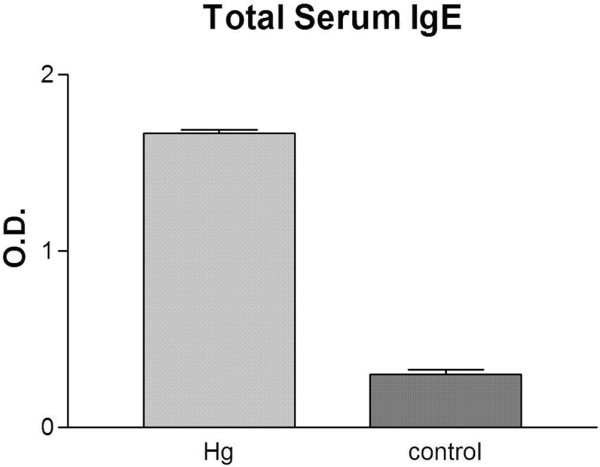
The effect of prior exposure to mercuric chloride (HgCl_2_) on serum IgE levels in *Chlamydia*-induced arthritis. The serum IgE levels are significantly increased compared with controls (*P *< 0.0001) (n = 8 in each group). O.D., optical density.

There was a dramatic elevation of serum IgE levels (Figure 3) in HgCl_2_-exposed animals (1.66 ± 0.05) compared with controls (0.30 ± 0.07), representing a 4.5-fold increase with HgCl_2 _exposure (*P *< 0.0001) (n = 8 in each group).

### Anti-*Chlamydia *antibodies

**Figure 4:**
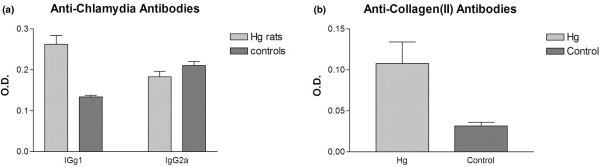
The effect of prior exposure to mercuric chloride (HgCl_2_) on serum anti-*Chlamydia *antibodies and anti-collagen antibodies in *Chlamydia*-induced arthritis. **(a) **HgCl_2_-exposed rats had significantly higher serum IgG1 anti-*Chlamydia *antibodies than non-exposed control rats (*P *< 0.001). Control rats had slightly higher IgG2a anti-*Chlamydia *antibodies than the HgCl_2_-exposed rats (*P *= 0.12). **(b) **HgCl_2_-exposed Brown Norway (BN) rats had higher levels of IgG antibodies to rat type II collagen than controls (*P *< 0.01) (n = 8 in each group). O.D., optical density.

The HgCl_2_-exposed rats (n = 8) had significantly higher serum IgG1 anti-*Chlamydia *antibodies (0.26 ± 0.05) than non-exposed control rats (0.13 ± 0.03) (*P *< 0.001) (Figure 4a). On the other hand, the control rats (n = 8) had slightly higher (*P *= 0.12) IgG2a anti-*Chlamydia *antibodies (0.21 ± 0.02) than the HgCl_2_-exposed rats (0.18 ± 0.03). The IgG2a to IgG1 ratio was 1.57 for the control rats in contrast to 0.70 for the HgCl_2_-exposed animals, consistent with a shift toward a Th2 response after HgCl_2 _exposure.

### Anti-collagen_(II) _antibodies

The HgCl_2_-exposed BN rats (n = 8) had higher levels of IgG antibodies to rat type II collagen than did controls: 0.108 ± 0.069 versus 0.032 ± 0.012 (*P *< 0.01). These serological changes paralleled the enhanced severity of arthritis clinically and histologically following HgCl_2 _exposure (Figure 4b).

### Cytokine profiles

**Figure 5:**
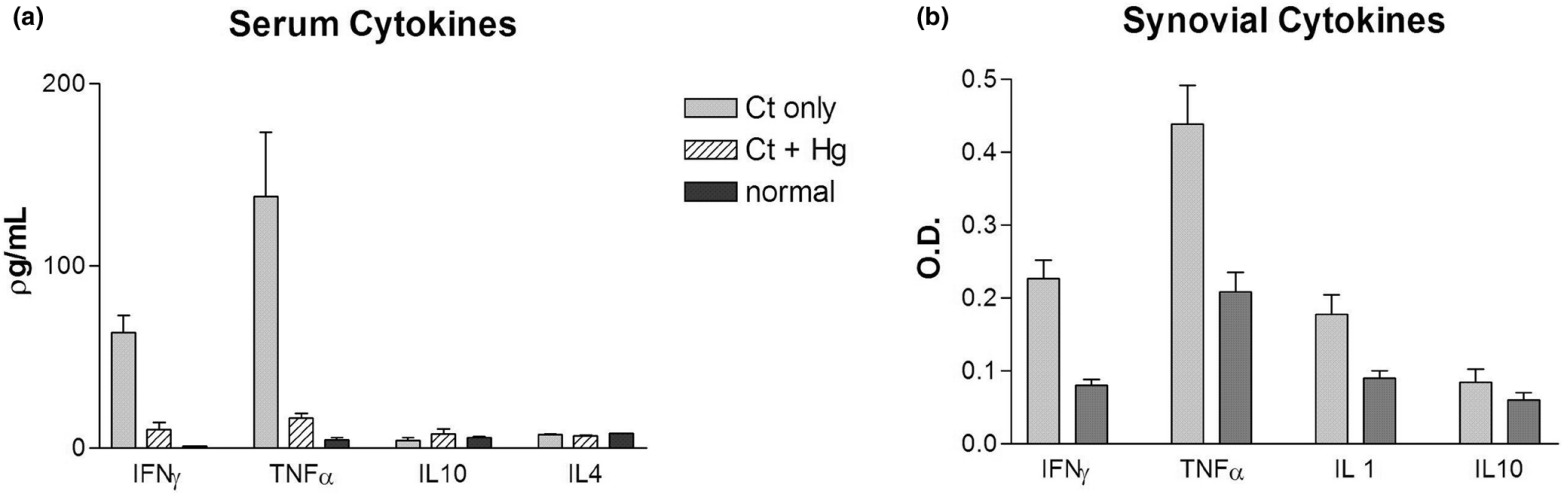
Serum and synovial cytokine profiles. **(a) **Serum cytokines in normal rats and in *Chlamydia*-induced arthritis alone or after prior exposure to mercuric chloride (HgCl_2_). After the onset of *Chlamydia trachomatis*-induced arthritis (CtIA), there was a significant increase in the serum levels of interferon-gamma (IFN-γ) (*P *< 0.005) and tumour necrosis factor-alpha (TNF-α) (*P *< 0.001). Prior HgCl_2 _exposure was associated with significant suppression of IFN-γ (*P *= 0.002) and TNF-α (*P *= 0.014) seen with CtIA. **(b) **Synovial cytokines in *Chlamydia*-induced arthritis alone or after prior exposure to HgCl_2_. HgCl_2 _exposure was accompanied by a decrease in local cytokine production for IFN-γ (*P *< 0.005), TNF-α (*P *< 0.05), and interleukin (IL)-1 (*P *< 0.05) compared with controls. No significant difference was observed for synovial IL-10 (n = 4 in each group).

After intra-articular injection with *Chlamydia*, there was an increase in the serum levels of IFN-γ (n = 4) and TNF-α (n = 4) (Figure 5a). These were significant increases compared with normal rat serum for both IFN-γ (*P *< 0.005) and TNF-α (*P *< 0.001). There were no significant increases in serum IL-10 or IL-4 after *Chlamydia *joint injection (n = 4). Prior HgCl_2 _exposure was associated with suppression of the expected rise in serum IFN-γ and TNF-α seen with CtIA. The differences between the HgCl_2_-exposed and non-exposed controls were significant for IFN-γ (*P *= 0.002) and TNF-α (*P *= 0.014). No comparable differences were detected for IL-4 or IL-10, but the baseline levels were low in both cases. Inflamed synovial tissues from *Chlamydia*-injected rats were harvested for assays for tissue cytokine profiles using the dot-blot method (Figure 5b). HgCl_2 _exposure was accompanied by a decrease in local cytokine production for IFN-γ (*P *< 0.005), TNF-α (*P *< 0.05), and IL-1 (*P *< 0.05). No significant difference was observed for synovial IL-10.

### *Chlamydia *clearance

**Figure 6:**
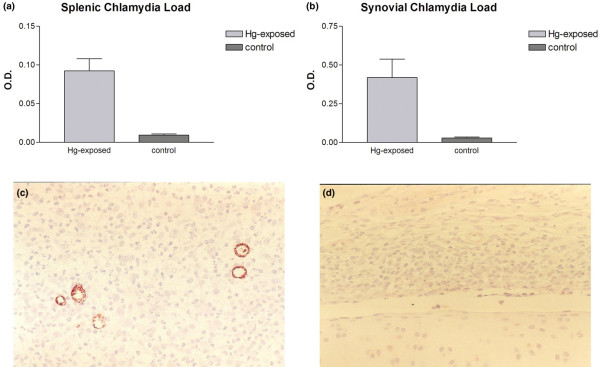
Host clearance of *Chlamydia *from the spleen and the joint. Tissues were harvested 5 days after the induction of *Chlamydia trachomatis*-induced arthritis (CtIA). **(a) **The mean level of the splenic bacterial load for the mercuric chloride (HgCl_2_)-exposed rats was higher than that of controls (*P *< 0.005). **(b) **In the joint, the mean *Chlamydia *load for the HgCl_2_-exposed rats was significantly higher than that of controls (*P *< 0.05). Immunohistology shows more intense staining with anti-*Chlamydia *antibodies in the HgCl_2_-exposed rats **(c) **than controls **(d) **(n = 5 in each group). Original magnifications × 200. O.D., optical density.

HgCl_2_-exposed rats demonstrated a relative defect in host clearance of the pathogen both locally and systemically (n = 5 in each group). The mean level of the splenic bacterial load (measured in optical density units) for the HgCl_2_-exposed rats was 0.092 ± 0.035 in contrast with 0.009 ± 0.003 for the controls (Figure 6a). This clearance difference is statistically significant (*P *< 0.005). In the joint, the average *Chlamydia *load for the HgCl_2_-exposed rats was significantly higher (0.419 ± 0.264) than that of controls (0.028 ± 0.014) (*P *< 0.05) (Figure 6b). Immunohistology shows more intense staining with anti-*Chlamydia *antibodies in the HgCl_2_-exposed rats (Figure 6c) than in controls (Figure 6d), reflecting the relative failure of the HgCl_2_-exposed rat to achieve local clearance of the pathogen.

### Nitric oxide and vascular endothelial growth factor

**Figure 7:**
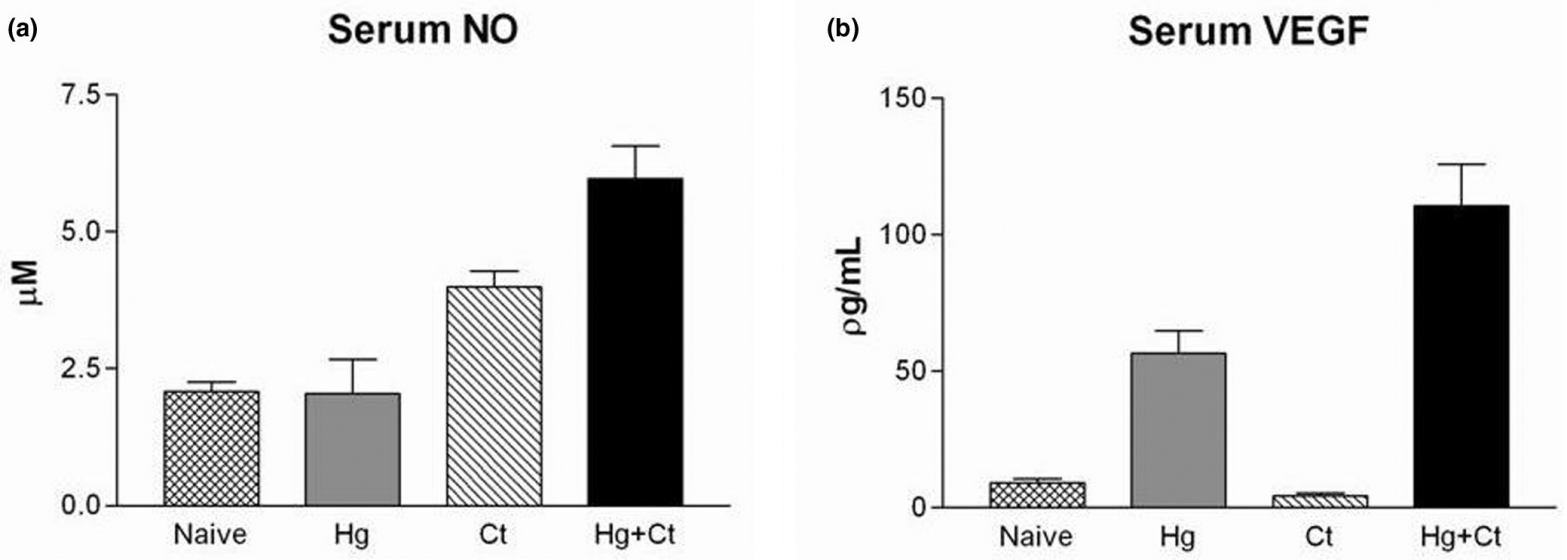
Serum nitric oxide and VEGF profiles. Serum nitric oxide (NO) levels **(a) **and vascular endothelial growth factor (VEGF) levels **(b) **with the local exposure to *Chlamydia trachomatis *(Ct) or a systemic exposure to HgCl_2 _(Hg) or both (Ct + Hg) in contrast to normal rat serum. For both NO and VEGF, the increment seen with dual-exposure to Ct and HgCl_2 _was statistically greater than that seen with either Ct or HgCl_2 _alone (n = 4 in each group). HgCl_2_, mercuric chloride.

Serum NO was elevated (3.99 ± 0.83 μM) in CtIA compared with serum from naïve rats (n = 4) (2.08 ± 0.47 μM). HgCl_2 _alone had no impact on serum NO compared with naïve rats, but there was an additive effect of Ct and HgCl_2 _observed (5.97 ± 1.58 μM; *P *< 0.001) (Figure 7a). Serum VEGF was increased by exposure to HgCl_2 _alone (56.67 ± 20.22 pg/mL) in comparison with normal rat serum (9.21 ± 3.3 pg/mL), but Ct alone did not elevate serum VEGF in comparison with normal rat serum (Figure 7b). In contrast, Ct and HgCl_2 _had an additive effect on the serum VEGF (110.56 ± 37.37 pg/mL; *P *< 0.001 in comparison with normal rat serum). Four animals were studied in each group. The elevation in NO and VEGF was in contrast to some of the cytokine-suppressive effects of HgCl_2 _exposure, with serum levels of NO and VEGF rising more with the combined exposure than to either exposure alone. This corresponded to the most intense pathological changes in the joints of animals with dual-exposure to both Ct and HgCl_2_.

## Discussion

The spondyloarthropathies (SpAs) refer to a group of diseases that share several common features: association with HLA class I genes, asymmetric oligoarthritis, axial involvement, enthesitis, and characteristic extra-articular features such as uveitis. The role of infection as a triggering factor is implicated with varying degrees of certainty amongst the SpA subcategories, with ReA having the most clear evidence for a microbial trigger [15]. The most direct causal evidence for microbial triggers for ReA derives from a demonstration of microbial antigens in the joint [16]. The case for intra-articular pathogens is strongest for *Chlamydia*, and several investigators have used polymerase chain reaction to demonstrate *Chlamydia *DNA or RNA in the joints of patients with post-*Chlamydia *ReA [17-19]. There is some evidence that these organisms are metabolically altered and may have entered a quiescent phase [20,21]. Several studies support the notion of a viable organism present, at least transiently, in the early stages of ReA. It is speculated that this may reflect defective killing of the organism by failure either to internalize the pathogen or to effectively initiate intracellular killing. There has been particular interest in the *Chlamydia *heat shock proteins, notably heat shock protein 60 (hsp60), and recent studies have identified differential expression of three *C. trachomatis *hsp60-encoding genes that may differ in active versus persistent infections [22]. The cellular response to *Chlamydia *infection has been studied by microarray techniques, and 18 genes appeared to be selectively upregulated following infection with *C. trachomatis *[23]. Profiling *Chlamydia *infection of U937 monocytic cells [24] and human lung epithelial cells [25] has provided a profile of induced gene expression and, in particular, which cytokines are induced by this microbial challenge.

Analysis of cytokine profiles is a further method for studying the link between infection and ReA. In a study of 11 patients with ReA, it was observed that stimulation of SF mononuclear cells resulted in secretion of low amounts of IFN-γ and TNF-α but high amounts of IL-10 [26]. IL-10 was responsible for suppression of IFN-γ and TNF-α as judged by the effect of adding IL-10 or anti-IL-10 to the cells. The suppression of Th1-like cytokines is likely mediated through suppression of IL-12 synthesis. This IL-10/IL-12 balance, resulting in a predominance of Th2 cytokines, may contribute to the persistence of bacteria in the joint. In comparison with rheumatoid arthritis, SF levels of TNF-α in ReA are lower despite comparable levels of IL-2 receptor, again implicating a relative deficiency of protective antimicrobial cytokines in the local environment [27]. Analysis of synovial fluid cytokines has been studied in patients with *Chlamydia*-induced arthritis and it was found that B27^+ ^patients had lower SF IFN-γ levels and it was these patients who had a more chronic course [28]. This suggests that diminished IFN-γ generation might account for the persistence of the arthritis.

Our previous studies in experimental ReA have paralleled these clinical findings. BN rats that are relatively resistant to CtIA exhibit an enhanced IFN-γ and TNF-α expression in the microenvironment of the joint. This is accompanied by enhanced clearance of the pathogen and a more transient and benign course of the arthritis. In the present study, we have found that this inherent resistance to ReA can be overcome by heavy metal exposure. Mercury exposure alters the cytokine profile, host clearance capability, and histopathological outcome from the resistant phenotype to the susceptible phenotype. Our findings contrast with the experience studying murine collagen-induced arthritis, in which prior exposure to HgCl_2 _did not influence the outcome of the arthritis, but exposure following the onset did have an aggravating effect on the arthritis [29].

If HgCl_2 _diminishes protective cytokines in the microenvironment of the joint, what is driving the aggressive inflammation and subsequent joint damage? Our study suggests that NO and VEGF are two important candidates. We have previously studied NO contribution to clearance of arthritogenic pathogens by synoviocytes and observed that in some instances there was an IFN-γ-mediated suppression of NO in such cells, suggesting a dynamic interaction between these two factors [30]. This interaction may be specific to the cells under investigation since, in the case of J774 macrophages, IFN-γ mediated an increase in NO production, which in turn reduced viability of *C. pneumoniae *in the infected cells [31]. In our *in vivo *model, the rise in NO was concurrent with an increase in chlamydial load in the host, suggesting that the increase in NO was not sufficient to contain an expanded replication profile of the organism. In this regard, it is interesting to note that, in murine cells, blockage of NO synthesis only partially rescues chlamydial growth, suggesting that there are other important IFN-γ-inducible antichlamydial mechanisms operative [32].

Prior studies on the effect of Hg on NO have yielded varying results depending on the experimental conditions. Kim and colleagues [33] found that Hg treatment of a macrophage cell line results in a drop in NO production upon stimulation with lipopolysaccharide. However, Huang and colleagues [34] discovered that treatment of mice with Hg resulted in an increase in serum NO levels. The latter results are very much in parallel with our finding of an increase of NO following exposure to HgCl_2_.

The pathological studies in our rats indicate that the exacerbation of histological severity of the arthritis was accompanied by a neovascularization process locally. It is known that VEGF plays a crucial role in angiogenesis. Spondylarthritis is characterized by enthesitis and synovitis, in which new blood vessels participate in perpetuating the inflammation. Serum analysis from SpA patients has documented that VEGF levels were significantly higher in SpA patients than in controls. In SpA patients, serum VEGF levels correlated with disease activity indices as defined by the Bath Ankylosing Spondylitis Disease Activity Index (BASDAI), erythrocyte sedimentation rate, or C-reactive protein (CRP). These results suggest that VEGF and therefore angiogenesis may play a role in SpA pathogenesis and may serve as a disease activity marker in SpAs [35]. In keeping with these findings, it has been observed that there is a significant reduction in serum VEGF levels after the infliximab treatment of SpA patients and that these changes correlate with similar reductions in CRP and IL-6 [36]. Direct *Chlamydia*-endothelial cell interactions are known to mediate induction of VEGF [37].

Mercury exists in elemental, inorganic, and organic forms. The general population is exposed primarily to mercury vapour from dental amalgam and to organic mercury from fish consumption [38]. Mercury accumulates in the food chain such that large fish such as tuna and swordfish have high concentrations of mercury in tissues [39]. Occupational exposure is primarily to mercury vapour and occurs in dentistry, mining, and the manufacture of electrical equipment. Mercury vapour is well absorbed through the respiratory tract, but absorption of elemental mercury is negligible orally whereas oral absorption of organic mercury is nearly complete [40]. There is recent evidence from studies of human peripheral mononuclear cells that low-dose exposure to mercury can polarize the immune response toward Th2 [41].

The role of mercury in rheumatic diseases has received little attention. One recent study implicated mercury exposure as a risk factor for Wegener granulomatosis [42], but the mechanism whereby mercury exposure could alter immune response and set the stage for chronic inflammatory conditions has not been resolved. Our experimental system demonstrates that genetic susceptibility can be fundamentally altered by a heavy metal exposure. This suggests that several environmental factors may act in concert in the pathogenesis of ReA. The immediate precipitating factor may be a bacterial infection, but this occurs in the context of other factors in the environment which influence the immune repertoire. The plasticity of the immune response, even when encoded in its basic elements by heritable factors, is highlighted in this genetic-environment interaction. As significant advances in the genetic basis of rheumatic diseases are being made, it will be a challenge to address the environmental factors with equal rigour.

## Conclusion

Genetically defined cytokine production in the joint defines the severity of ReA by dictating the local clearance of the pathogen. This interplay can be dramatically altered by heavy metal exposure, which results in suppression of protective cytokines in the microenvironment of the joint.

## Abbreviations

BN: Brown Norway; CFU: colony-forming units; CRP: C-reactive protein; Ct: *Chlamydia trachomatis*; CtIA: *Chlamydia trachomatis*-induced arthritis; ELISA: enzyme-linked immunosorbent assay; Hg: mercury; HgCl_2_: mercuric chloride; hsp60: heat shock protein 60; IFN-γ: interferon-gamma; IL: interleukin; NO: nitric oxide; PBS: phosphate-buffered saline; ReA: reactive arthritis; SF: synovial fibroblast; SpA: spondyloarthropathy; TNF-α: tumour necrosis factor-alpha; VEGF: vascular endothelial growth factor.

## Competing interests

The authors declare that they have no competing interests.

## Authors' contributions

RDI and BC both contributed to the design and execution of the study, to the data analysis, and to the writing of the manuscript. Both authors read and approved the final manuscript.
